# Auto-Regulation of the *Sohlh1* Gene by the SOHLH2/SOHLH1/SP1 Complex: Implications for Early Spermatogenesis and Oogenesis

**DOI:** 10.1371/journal.pone.0101681

**Published:** 2014-07-08

**Authors:** Shuichi Toyoda, Takuji Yoshimura, Junya Mizuta, Jun-ichi Miyazaki

**Affiliations:** 1 Division of Stem Cell Regulation Research, Osaka University Graduate School of Medicine, Osaka, Japan; 2 Laboratory of Reproductive Engineering, the Institute of Experimental Animal Sciences, Osaka University Medical School, Osaka, Japan; University of Saarland Medical School, Germany

## Abstract

Tissue-specific basic helix-loop-helix (bHLH) transcription factor proteins often play essential roles in cellular differentiation. The bHLH proteins SOHLH2 and SOHLH1 are expressed specifically in spermatogonia and oocytes and are required for early spermatogonial and oocyte differentiation. We previously reported that knocking out *Sohlh2* causes defects in spermatogenesis and oogenesis similar to those in *Sohlh1*-null mice, and that *Sohlh1* is downregulated in the gonads of *Sohlh2*-null mice. We also demonstrated that SOHLH2 and SOHLH1 can form a heterodimer. These observations led us to hypothesize that the SOHLH2/SOHLH1 heterodimer regulates the *Sohlh1* promoter. Here, we show that SOHLH2 and SOHLH1 synergistically upregulate the *Sohlh1* gene through E-boxes upstream of the *Sohlh1* promoter. Interestingly, we identified an SP1-binding sequence, called a GC-box, adjacent to these E-boxes, and found that SOHLH1 could bind to SP1. Furthermore, chromatin-immunoprecipitation analysis using testes from mice on postnatal day 8 showed that SOHLH1 and SP1 bind to the *Sohlh1* promoter region *in vivo*. Our findings suggest that an SOHLH2/SOHLH1/SP1 ternary complex autonomously and cooperatively regulates *Sohlh1* gene transcription through juxtaposed E- and GC-boxes during early spermatogenesis and oogenesis.

## Introduction

Transcriptional regulation is essential for cellular differentiation. Previous studies have demonstrated that a number of transcriptional factors play important roles in early spermatogenesis and oogenesis [1], [2]. Recently, several gene-knockout studies revealed that the germ cell-specific basic helix-loop-helix (bHLH) proteins SOHLH2 and SOHLH1 are expressed in spermatogonia and early oocytes [3]–[6] and are required for their differentiation [4]–[8]. The *Sohlh2* transcript is upregulated shortly after birth, and the SOHLH2 protein is expressed in the adult testis by a portion of A_s_ spermatogonia throughout differentiation [6]. In mouse oogenesis, the *Sohlh2* transcript is upregulated before birth [6], and its protein is expressed in primordial through primary oocytes in the ovary [3], [6]. Although the *Sohlh1* and *Sohlh2* expression patterns are similar, the *Sohlh1* transcript is upregulated following *Sohlh2* expression in both early spermatogenesis and oogenesis [6]. The SOHLH1 protein is expressed by A_al_ spermatogonia throughout differentiation [3]. Since both male and female *Sohlh2-* and *Sohlh1*-null mice are infertile, and these mice have similar abnormalities in gonad histology and gene expression patterns, *Sohlh2* may be upstream to *Sohlh1* in the gene regulatory hierarchy.

The bHLH proteins are known to form heterodimers or homodimers to bind to the consensus E-box DNA sequence CANNTG. Some bHLH proteins, such as ARNT, can transactivate target genes through homodimerization [9], while others, such as MAX-MYC, transactivate their target genes through heterodimerization [10], [11]. It has been reported that SOHLH2 and SOHLH1 can form a heterodimer [6], and that the *Sohlh1* mRNA levels are significantly reduced in the *Sohlh2*-null testis and ovary compared to the levels in wild-type gonads [6]–[8]. Since *Sohlh1* contains several E-box (CACGTG) motifs in its promoter region (see below), it is possible that the SOHLH proteins regulate the *Sohlh1* gene.

Transcription factors often function by forming complexes with other proteins. The bHLH proteins sometimes form ternary complexes with SP1, which is a zinc finger-type transcription factor [12] that binds to the consensus DNA sequence GGGGCGGGGC, called a GC-box [13]. The ternary complex binds to juxtaposed E- and GC-boxes and synergistically transactivates the adjacent promoter, as seen in Myogenin/SP1 and NeuroD1/SP1 complexes [14], [15]. SP1 is widely expressed in various cell types, including spermatogonia and oocytes [16], [17]. Interestingly, here we identified juxtaposed E- and GC-box sequences in the upstream region of the *Sohlh1* gene of various mammalian species, and found evidence that the SOHLH proteins form a ternary complex with SP1 to regulate the *Sohlh1* gene. We also identified the motifs in the *Sohlh1* promoter involved in this regulation. These findings improve our understanding of the molecular mechanisms that regulate *Sohlh1* in male and female germ-cell differentiation.

## Materials and Methods

### Ethics statement

Experiments involving animals were carried out in accordance with institutional guidelines under protocols (No. 21–089) approved by the Animal Care and Use Committee of the Osaka University Graduate School of Medicine.

### Cell culture

HEK293 cells (BioWhittaker, Walkersville, MD) were cultured in Minimum Essential Medium (Sigma-Aldrich, St. Louis, MO; Cat#M0643) supplemented with 10% heat-inactivated fetal calf serum, at 37°C.

### Western blotting and immunoprecipitation assay

Samples were homogenized in RIPA buffer (10 mM Tris-HCl, pH 7.4, 1 mM EDTA, 150 mM NaCl, 1% NP-40, 0.1% SDS, and 0.1% sodium deoxycholate). The extracted protein was mixed 2∶1 v/v with 3x sample buffer (New England BioLabs, Beverly, MA; Cat#B7703S) and a 1/30 volume of 1.25 M dithiothreitol (DTT), after which it was heated at 99°C for 5 min, separated in SDS-polyacrylamide gel, and analyzed by western blotting as described previously [18]. The following primary antibodies were used in this study: mouse anti-FLAG antibody (Sigma-Aldrich; Cat#F3165, 2 µg/ml at final concentration), rabbit anti-Myc-tag antibody (MBL, Nagoya, Japan; Cat#562, 1∶1000 dilution), rabbit anti-SOHLH2 antibody [6], rabbit anti-SOHLH1 antibody (Abcam, Cambridge, MA; Cat#ab49272, 1∶5000 dilution), and rabbit anti-SP1 antibody (Bethyl Laboratories, Montgomery, TX; Cat#IHC-00208, 1∶200 dilution). Secondary antibodies used in this study were as described previously [6]. An immunoprecipitation assay was performed as described previously, using agarose beads conjugated with an anti-FLAG antibody (Sigma-Aldrich; ANTI-FLAG M2 Affinity Gel, Cat#F2426) [6].

### Vector construction

The promoter region of the mouse *Sohlh1* gene (−1036 to −1 bp upstream of the *Sohlh1* translational start site) was obtained by PCR from the genomic DNA of E14, a mouse embryonic stem cell line derived from a 129/Ola mouse strain. For E- and GC-box mutagenesis, we used a PCR-based method using primers with mutated sequences. Promoters containing a deletion were prepared by PCR or with the appropriate restriction enzymes. These promoters were inserted into the multi-cloning sites of the pGL3-Basic vector (Promega, Madison, WI; Cat#E1751) and used for reporter assays. The pCMV-FLAG-Sohlh1, pCMV-FLAG-Sohlh2, pCAG-Sohlh1, and pCAG-Sohlh2 plasmid vectors were constructed as described previously [6]. The pcDNA3-Sohlh2-Myc and pcDNA3-Sohlh1-Myc vectors were obtained by inserting mouse *Sohlh2* and *Sohlh1* cDNA, respectively, into the pcDNA3-Myc-His vector (Invitrogen, Carlsbad, CA; Cat#V855-20). Mouse *Sp1* cDNA obtained from testis RNA by reverse transcription followed by PCR was inserted into a pCAG-IP plasmid vector [19]. All the PCR-amplified fragments were confirmed by sequencing. The primers used in this study are available upon request.

### Reporter assays

HEK293 cells were plated on 24-well plates at a density of 2×10^4^ cells per well, 24 hours prior to transfection. The cells were then co-transfected with 200 ng of a reporter vector, 0.32 to 200 ng of expression vectors, and 0.1 ng of pRL-CMV normalization vector per well using HilyMax (Dojindo Molecular Technologies, Kumamoto, Japan; Cat#H357–10). After 48 hours, the total cell extracts were obtained and subjected to luciferase assays using the Dual-Luciferase Reporter Assay System (Promega).

### Chromatin-immunoprecipitation (ChIP) assay

Testes were isolated from three wild-type mice on postnatal day (P) 8 and were fixed in 500 µl of fixation buffer (1% formaldehyde, 4.5 mM HEPES, 9 mM NaCl, 0.09 mM EDTA) for 10 min at room temperature followed by adding 55 µl of 1.5 M glycine to stop the crosslinking reaction. ChIP experiments were performed using the EZ ChIP kit (Millipore, Billerica, MA; Cat#17–371) according to the manufacture's instruction. After washing three times with 1 ml of ice-cold phosphate-buffered saline (PBS), testicular cells were lyzed in 400 µl of SDS lysis buffer and sonicated with a sonicator (Branson, Danbury, CT). After centrifugation at 18,000 *g* for 5 min, 50 µl of the supernatant was diluted with 450 µl ChIP dilution buffer containing 0.5% protease inhibitor cocktail. Magna beads and rabbit anti-SOHLH1 antibody (Abcam), rabbit anti-SP1 (ChIPAb+ Sp1, Millipore; Cat#17–601) antibody, or normal rabbit IgG were added to the samples, and incubated overnight at 4°C. Then, the samples were washed once with Low Salt Immune Complex Wash Buffer, once with High Salt Immune Complex Wash Buffer, once with LiCl Immune Complex Wash Buffer, and twice with TE buffer. The precipitated DNA was liberated from the immune complex by adding 100 µl of ChIP Elution Buffer and 1 µl of Protenase K followed by heating at 62°C for 2.5 hours. DNA was recovered using Spin filter column, eluted in 100 µl of TE buffer, and applied to qPCR. Genomic regions upstream of the *Sohlh1* gene were amplified using specific primer pairs: 5′-TGCCCCTAGAAATCCACTAGAGACG-3′ and 5′-GATAGCTTGCAGCTCTGTTTCTGAC-3′ for the *Sohlh1* promoter region (−371 to −284); 5′-TGACACTGTCCACAACAGGAAGGAC-3′ and 5′-ATCCAGGCTGCCTTTCACTTTCTGC-3′ for a control region far upstream of the *Sohlh1* promoter (−8946 to −8834). Accumulation of fluorescent products was monitored using the StepOnePlus Real-Time PCR System (Applied Biosystems, Foster, CA).

## Results

### SOHLH2 and SOHLH1 form homodimers

**Figure 1:**
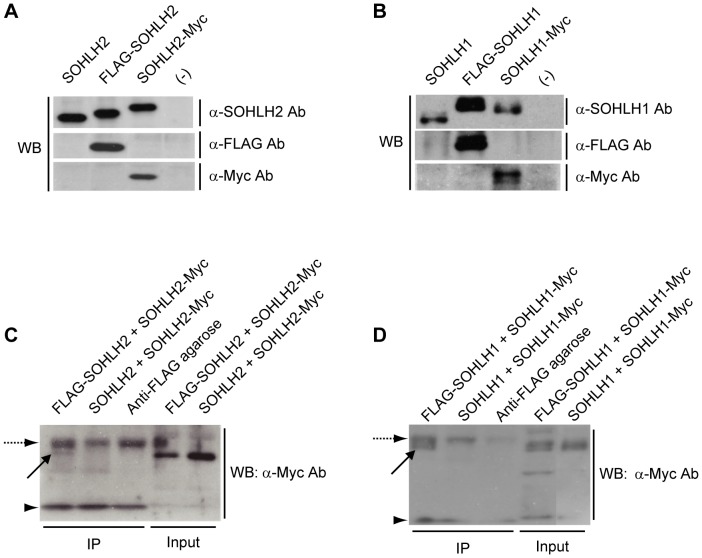
SOHLH2 and SOHLH1 form homodimers. (A) Western blots of lysates of HEK293 cells overexpressing SOHLH2, FLAG-SOHLH2, and SOHLH2-Myc, using anti-SOHLH2, anti-FLAG, and anti-Myc antibodies. (B) Western blots of lysates of HEK293 cells overexpressing SOHLH1, FLAG-SOHLH1, and SOHLH1-Myc, using anti-SOHLH1, anti-FLAG, and anti-Myc antibodies. (C) Lysates of HEK293 cells overexpressing FLAG-SOHLH2, SOHLH2-Myc, or SOHLH2 were immunoprecipitated with anti-FLAG agarose beads and subjected to western blotting using an anti-Myc antibody. Arrow: an SOHLH2-Myc band. Pre-immunoprecipitation lysates were used as input. (D) Lysates of HEK293 cells overexpressing FLAG-SOHLH1, SOHLH1-Myc, or SOHLH1 were immunoprecipitated with anti-FLAG agarose beads and subjected to western blotting using an anti-Myc antibody. Arrow: an SOHLH1-Myc band. Pre-immunoprecipitation lysates were used as input. Anti-FLAG agarose was loaded to indicate the IgG heavy chain (dashed arrow) and IgG light chain (arrowhead) bands.

Previously, we demonstrated that SOHLH2 forms a heterodimer with SOHLH1 [6]. To determine whether SOHLH2 and SOHLH1 can also form homodimers, we transiently co-expressed FLAG-SOHLH2 and FLAG-SOHLH1 with SOHLH2-Myc and SOHLH1-Myc, respectively, in HEK293 cells, which endogenously express neither SOHLH2 nor SOHLH1 (**Figure 1A and 1B**). Control western blot experiments shown in **Figure 1A and 1B** confirmed that the anti-FLAG antibody did not cross-react with SOHLH2-Myc or SOHLH1-Myc, and that the anti-Myc antibody did not cross-react with FLAG-SOHLH2 or FLAG-SOHLH1. Immunoprecipitation-assay bands indicating homodimerization were detected for both SOHLH2 (**Figure 1C** **, lane 1**) and SOHLH1 (**Figure 1D** **, lane 1**). These observations suggested that SOHLH2 and SOHLH1 could form homodimers *in vivo*, in agreement with another recent report that SOHLH2 and SOHLH1 form both heterodimers and homodimers [20].

### The SOHLH2/SOHLH1 heterodimer upregulates the *Sohlh1* promoter

**Figure 2:**
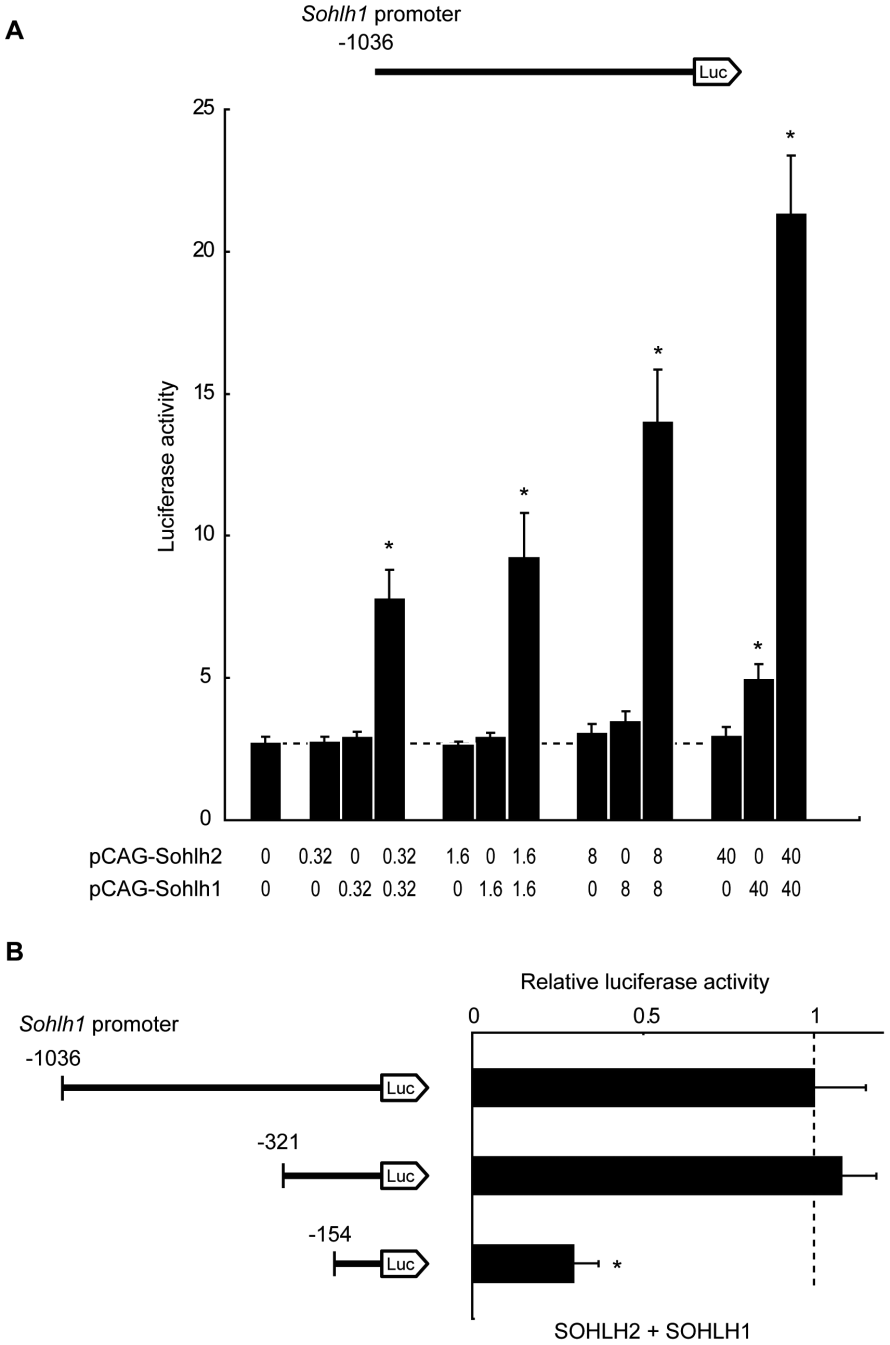
SOHLH2/SOHLH1 heterodimer regulates the *Sohlh1* promoter through a restricted upstream region. (A) Reporter assay using the pGL3-Basic vector containing the −1036 bp mouse *Sohlh1* promoter (200 ng), a pCAG-Sohlh2 expression vector (0 to 40 ng), a pCAG-Sohlh1 expression vector (0 to 40 ng), and a pRL-CMV normalization vector (0.1 ng). Results show the *Sohlh1* promoter-driven firefly luciferase activity relative to CMV promoter-driven Renilla luciferase activity. (B) Reporter assays using pGL3-Basic vectors containing various lengths of the mouse *Sohlh1* promoter (200 ng), a pCAG-Sohlh2 expression vector (200 ng), a pCAG-Sohlh1 expression vector (200 ng), and a pRL-CMV normalization vector (0.1 ng). Results show the firefly/Renilla luciferase activity relative to that obtained with the −1036 bp promoter fragment, which was arbitrarily set at 1. Error bars represent the S.E.M. of the means of 3–5 separate experiments done in triplicate. *P* values were calculated by Student's *t*-test. *P<0.05. SOHLH2 and SOHLH1 regulate the activity of the mouse *Sohlh1* promoter through a region −154 to −321 bp upstream from its translational start site.

During testicular and ovarian development, *Sohlh2* mRNA is upregulated prior to *Sohlh1* expression. The *Sohlh1* expression in *Sohlh2*-null mice remains low in the testis or ovary [6]. These observations indicated that the SOHLH2 protein might regulate *Sohlh1* gene activity by forming a homodimer or a heterodimer with SOHLH1. To evaluate the roles of SOHLH2 and SOHLH1 in regulating the *Sohlh1* promoter activity, we introduced a luciferase reporter plasmid vector containing the 1036-bp promoter region of the mouse *Sohlh1* gene, along with various amounts of plasmid vectors expressing SOHLH2 or SOHLH1, into HEK293 cells. As shown in **Figure 2A**, SOHLH2 or SOHLH1 alone did not markedly transactivate the *Sohlh1* promoter more strongly than the reporter alone. However, introducing the reporter plasmid with 0.32 ng, 1.6 ng, 8 ng, or 40 ng each of SOHLH2 and SOHLH1 expression plasmids significantly increased the reporter gene expression 2.8-, 3.4-, 5.1-, and 7.8-fold, respectively, relative to that of the reporter plasmid alone (**Figure 2A**). Thus, co-expressing SOHLH2 and SOHLH1 caused a dose-dependent increase in *Sohlh1* promoter activity.

### Species-conserved E-boxes in the *Sohlh1* promoter are important for transactivation

To determine which sequences in the *Sohlh1* gene promoter are required for its transcriptional activation by SOHLH2 and SOHLH1, HEK293 cells were transfected with reporter-gene plasmids containing various lengths of the 5′ upstream sequence of the *Sohlh1* promoter, along with 40 ng each of SOHLH2- and SOHLH1-expression plasmid vectors. Reporter plasmids containing the 1036-bp and 321-bp upstream sequences of the *Sohlh1* gene produced comparable luciferase activity (**Figure 2B**). However, a reporter plasmid containing the 154-bp upstream region of the *Sohlh1* gene showed 70% less luciferase activity, indicating that the region from −321 to −154 of the *Sohlh1* promoter contains important sequences for *Sohlh1*'s regulation by SOHLH2 and SOHLH1.

**Figure 3:**
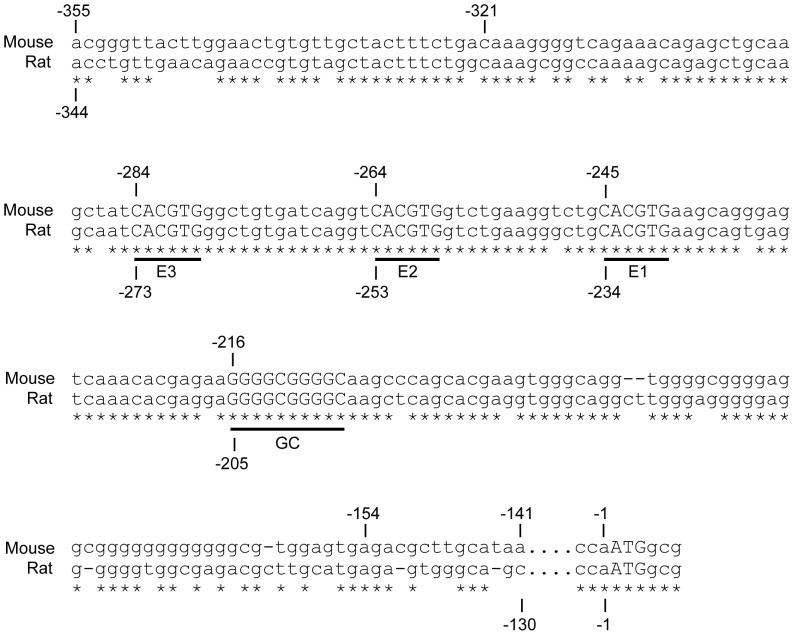
Conserved regulatory regions of the mouse and rat *Sohlh1* gene. The underlined E- and GC-box sequences are conserved between the mouse and rat. These E- and GC-box sequences are also found in the *Sohlh1* promoter region of the chimpanzee and human (not shown).

Since conservation between species often highlights important functional sequences, we analyzed sequences in the publicly available NCBI genomic database (http://www.ncbi.nlm.nih.gov/). We found that the mouse *Sohlh1* gene contains three E-boxes (CACGTG) from −240 bp to −284 bp upstream of its coding region, and that these sequences are well conserved in the rat. This conservation suggested that the SOHLH proteins might regulate the *Sohlh1* gene through these E-boxes, which were designated E1, E2, and E3 (proximal, middle, and distal, respectively) (**Figure 3**).

### The E-boxes are not equal in regulating the *Sohlh1* gene

**Figure 4:**
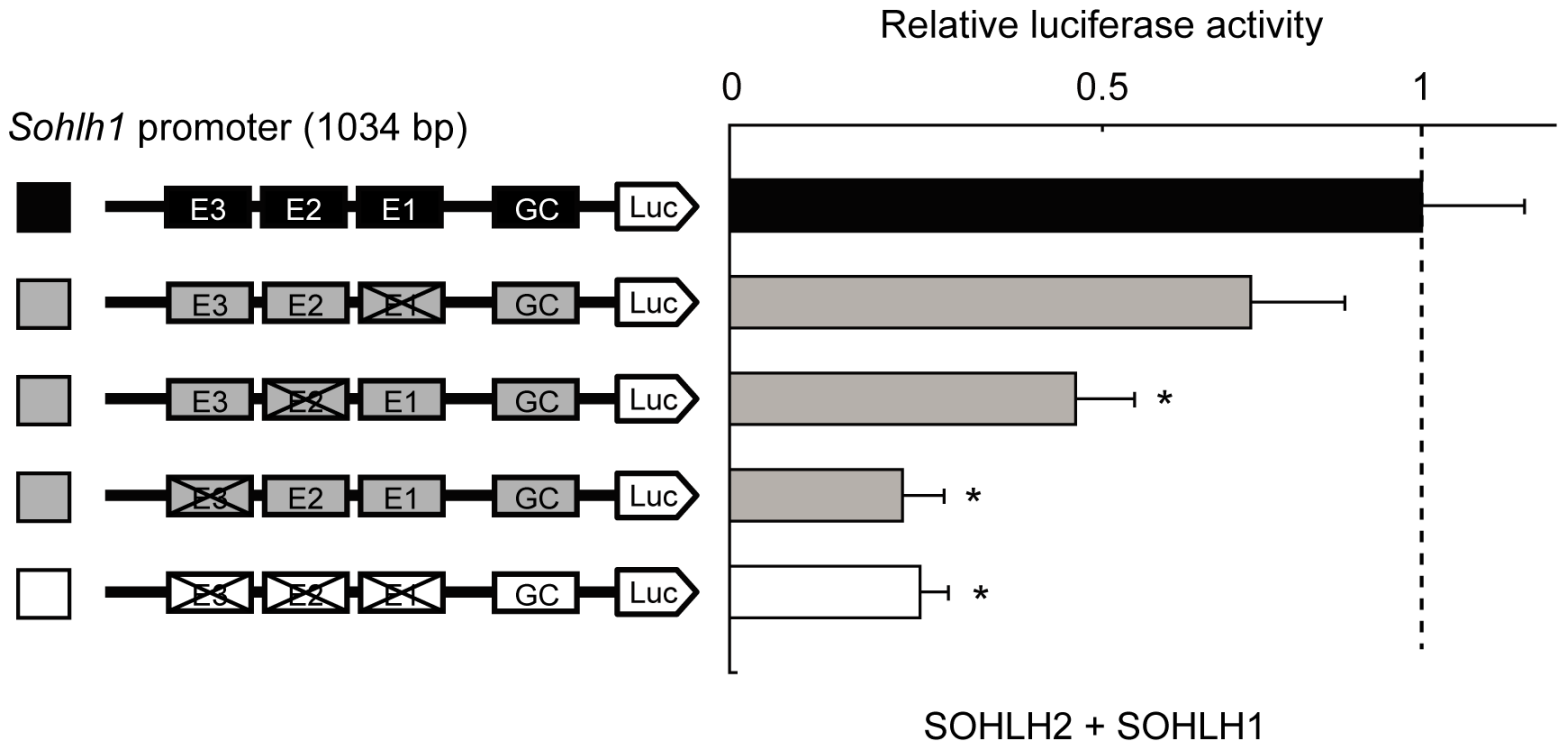
The three *Sohlh1*-promoter E-boxes are not equal in regulating *Sohlh1*. Reporter assays using the pGL3-Basic vector with various mutations of the E-boxes in the *Sohlh1* promoter, along with pCAG-Sohlh1 and pCAG-Sohlh2 expression vectors (200 ng each). Results show the firefly/Renilla luciferase activity relative to that of the −1036 bp intact promoter, which was arbitrarily set at 1. Error bars represent the S.E.M. of the means of 3–6 separate experiments done in triplicate. *P* values were calculated by Student's *t*-test. *P<0.05.

To determine whether the SOHLH2/SOHLH1 heterodimer regulates the *Sohlh1* gene through these E-boxes, we constructed *Sohlh1* promoters containing mutations in specific E-boxes (CACGTG to GGATCC) and expressed them with SOHLH2 and SOHLH1 in reporter assays. While the promoter with an E1 or E2 mutation showed approximately 25% and 50% less luciferase activity, respectively, compared with the intact promoter, the E3-mutant promoter showed approximately 75% less activity (**Figure 4**). A comparable reduction was seen with an E1/E2/E3 triple-mutant promoter. These observations suggested that the E3 box was the most important site of SOHLH2/SOHLH1 heterodimer binding.

### SOHLH2, SOHLH1, and SP1 are functionally associated in *Sohlh1* promoter activation

While searching for conserved sequences in the region from −321 to −154 bp upstream of the *Sohlh1* gene, we also found a species-conserved GC-box (GGGGCGGGGC), which contains the binding sequence of the widely expressed transcription factor SP1, neighboring the E-boxes (**Figure 3**). Some bHLH proteins interact with SP1 to cooperatively activate target genes by binding to juxtaposed E- and GC-boxes [14], [15]. Therefore, it was possible that the SOHLH proteins cooperate with SP1 to activate *Sohlh1* through E- and GC-boxes in its promoter region.

**Figure 5:**
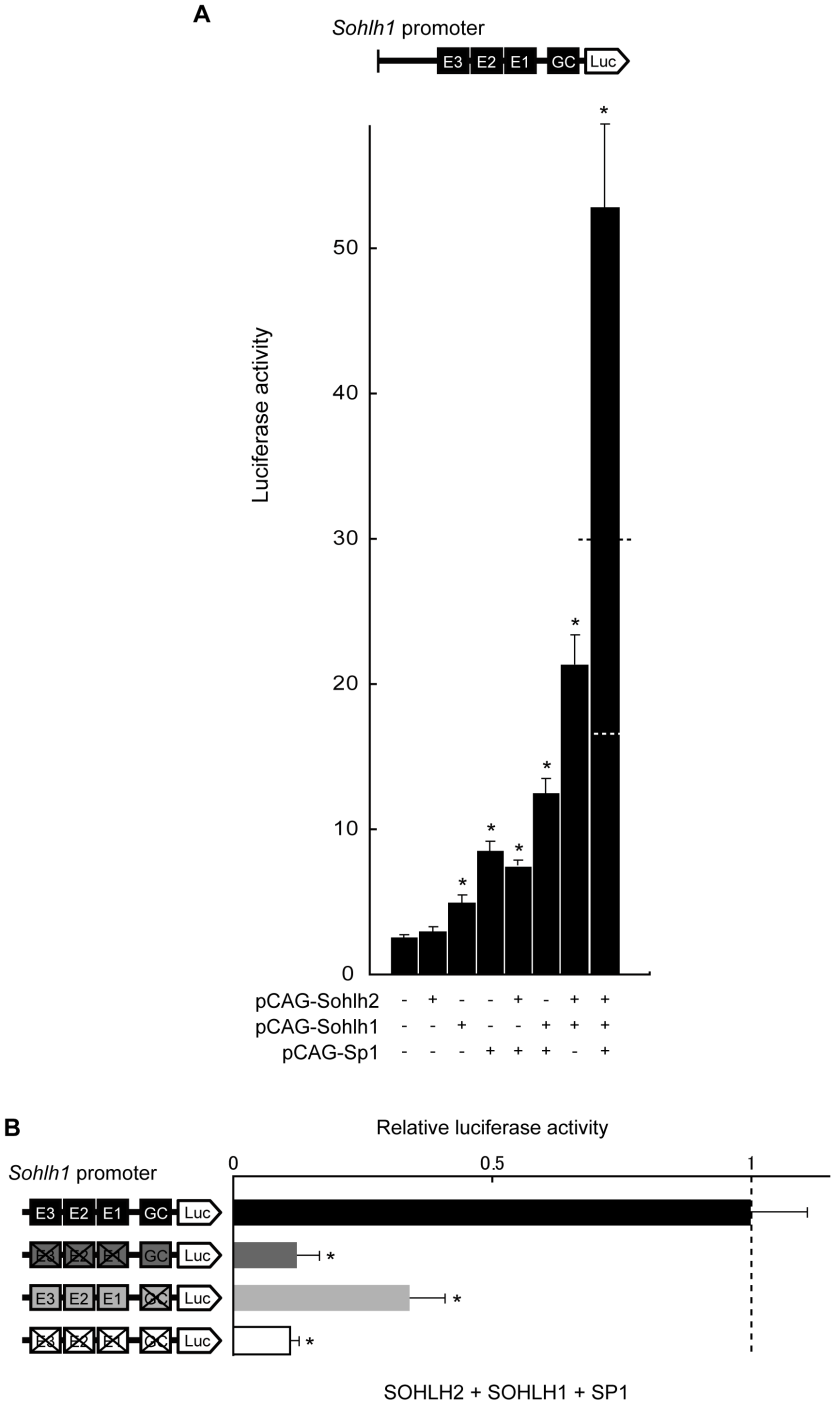
Transcriptional synergy between SP1 and the SOHLH proteins. (A) Reporter assays using the pGL3-Basic vector with the −1036 bp intact *Sohlh1* promoter (200 ng), a pRL-CMV normalization vector (0.1 ng), and expression vectors (pCAG-Sohlh2 (40 ng), pCAG-Sohlh1 (40 ng), and pCAG-Sp1 (40 ng)). Results show the *Sohlh1* promoter-driven firefly luciferase activity relative to that of CMV promoter-driven Renilla luciferase. The white dashed line indicates the sum of the individual transcriptional activities of SOHLH2, SOHLH1, and SP1. The black dashed line indicates the sum of the transcriptional activities of SOHLH2+SOHLH1 and SP1. *P* values were calculated by Mann-Whitney test. *P<0.05. (B) Reporter assays using the pGL3-Basic vector containing the −1036 bp *Sohlh1* promoter with various mutations in the E-boxes and/or GC-box (200 ng), a pRL-CMV normalization vector (0.1 ng), and expression vectors (pCAG-Sohlh2 (40 ng), pCAG-Sohlh1 (40 ng), and pCAG-Sp1 (40 ng)). Results show the firefly/Renilla luciferase activity relative to that of the intact −1036 bp *Sohlh1* promoter (black bar), which was arbitrarily set at 1. *P* values were calculated by Welch's *t*-test. *P<0.05. Error bars represent the S.E.M. of the means of 3–8 separate experiments done in triplicate.

To investigate the importance of the GC-box, we introduced the reporter vector containing the *Sohlh1* promoter and expression plasmid vectors for SOHLH2, SOHLH1, or SP1, alone or in combination, into HEK293 cells. Expressing SOHLH2, SOHLH1, or SP1 alone with the wild-type *Sohlh1* promoter vector increased the reporter activity by1.1-, 1.8-, or 2.9-fold above the basal level (the level of the reporter vector alone) (**Figure 5A**). Expressing SOHLH2 and SP1 or SOHLH1 and SP1 with the wild-type *Sohlh1* promoter vector enhanced the reporter activity by 2.5-, or 4.0-fold above the basal level, respectively (**Figure 5A**). However, expressing SOHLH2, SOHLH1, and SP1 together increased the reporter activity by approximately 19.2-fold above the basal level, far exceeding the sum of the activation levels obtained with each factor individually (**Figure 5A**). Thus, SOHLH2, SOHLH1, and SP1 transactivated the *Sohlh1* promoter synergistically.

We next examined whether this synergistic transcriptional activation of the *Sohlh1* gene by SOHLH2, SOHLH1, and SP1 required the binding sites we had postulated for these factors. In addition to the promoter with mutations in all three E-boxes, we produced an *Sohlh1* promoter with a mutation in the GC-box (GGGGCGGGGC to GAAGCTTGTC). We introduced these E-box or GC-box mutants, alone or in a combined, double-mutant reporter plasmid, into HEK293 cells along with SOHLH2, SOHLH1, and SP1 expression vectors (**Figure 5B**), and found that reporter activity decreased significantly, to 12.5%, 34.1%, and 10.9% of the activity of the intact promoter, with the E-box, GC-box, and double E- and GC-box mutations, respectively. These observations suggested that the SOHLH proteins, in cooperation with SP1, transactivate *Sohlh1* through juxtaposed E- and GC-boxes.

### SOHLH1 binds to SP1

**Figure 6:**
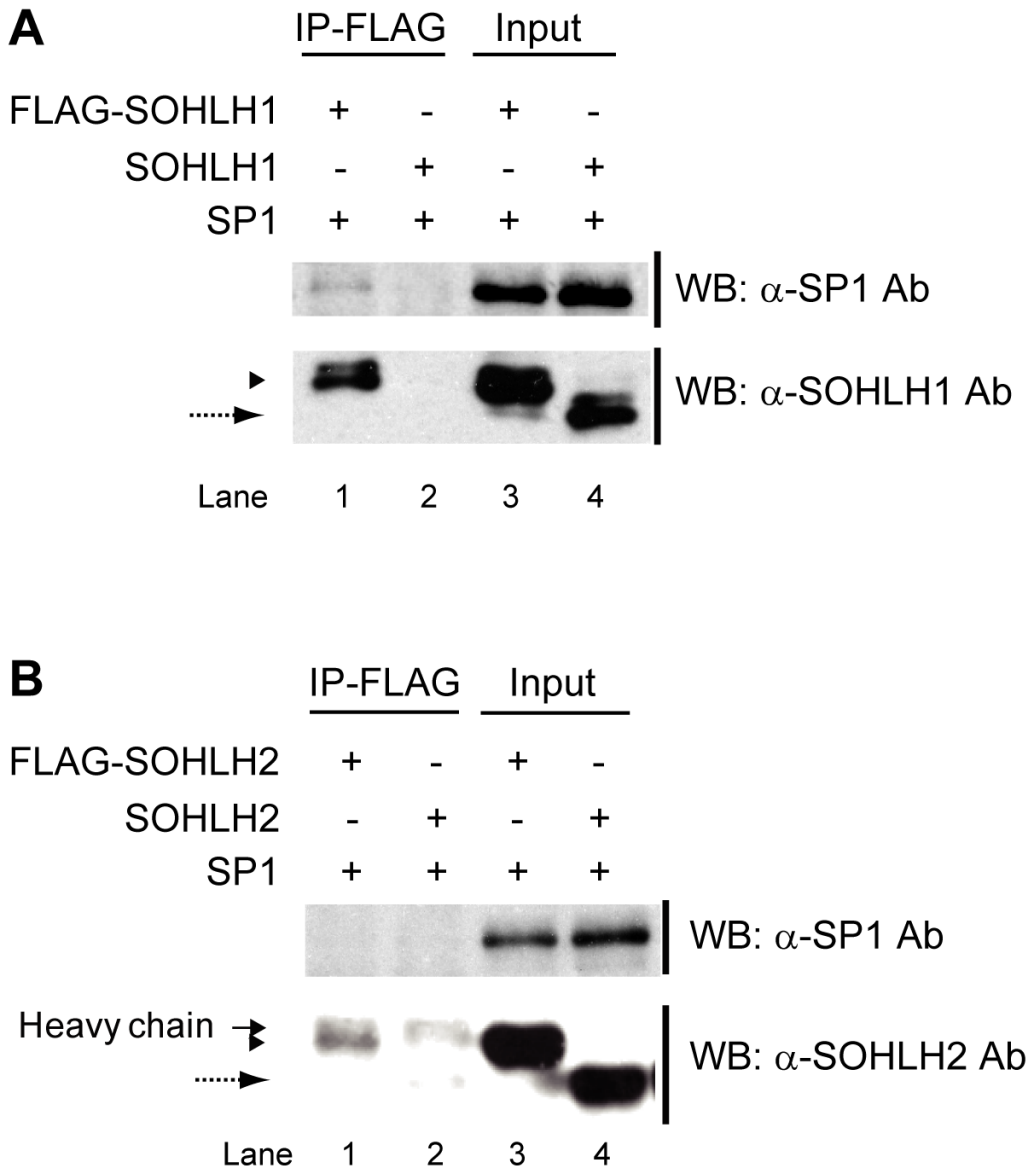
Interaction of SP1 to the SOHLH proteins. (A) Lysates of HEK293 cells overexpressing SP1 and FLAG-SOHLH1 or SOHLH1 were immunoprecipitated with anti-FLAG agarose beads and analyzed by western blotting using anti-SOHLH1 or anti-SP1 antibodies. Arrowhead: FLAG-SOHLH1. Dashed arrow: SOHLH1. (B) Lysates of HEK293 cells overexpressing SP1 and FLAG-SOHLH2 or SOHLH2 were immunoprecipitated with anti-FLAG agarose beads and analyzed by western blotting using anti-SOHLH2 or anti-SP1 antibodies. Arrowhead: FLAG-SOHLH2. Dashed arrow: SOHLH2. Arrow: IgG heavy chain. Pre-immunoprecipitation lysate was used as input.

To determine whether SOHLH proteins interact physically with SP1, we co-expressed SP1 and FLAG-tagged SOHLH1 or SOHLH2 in HEK293 cells. The FLAG-tagged SOHLH proteins immunoprecipitated from cell lysates with an anti-FLAG antibody were then analyzed by western blotting with an anti-SP1 antibody. These experiments showed that FLAG-SOHLH1 could associate with co-expressed SP1 (**Figure 6A**). On the other hand, the co-expression of FLAG-SOHLH2 and SP1 did not reveal any detectable association between SOHLH2 and SP1 (**Figure 6B**). Considering the heterodimerization of SOHLH2 and SOHLH1, SOHLH1 might act as a bridge between SOHLH2 and SP1, resulting in the formation of the SOHLH2/SOHLH1/SP1 complex. As it was reported that the DNA-binding domain of SP1 and the HLH domain of MYOGENIN or NEUROD1 mediate protein-protein interactions [14], [15], it is possible that the HLH domain of SOHLH1 also associates with SP1's DNA-binding domain.

### SOHLH1 and SP1 are recruited to the *Sohlh1* promoter region *in vivo*

**Figure 7:**
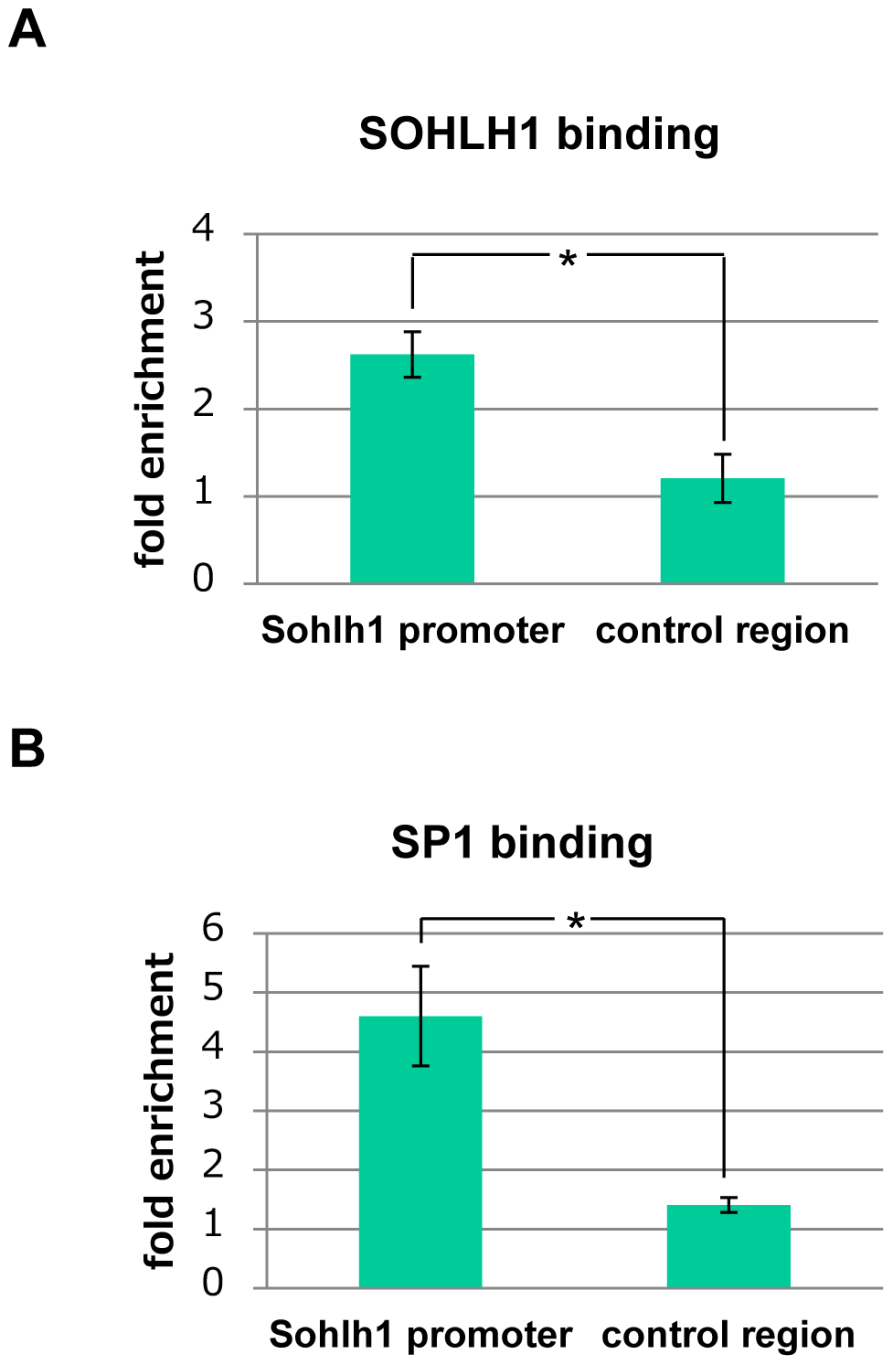
Binding of SOHLH1 and SP1 to the *Sohlh1* promoter region *in vivo*. Binding of SOHLH1 and SP1 to the *Sohlh1* promoter region *in vivo* was examined by ChIP assay using P8 testes. The *Sohlh1* promoter region (−371 to −284) and the control region far upstream of the *Sohlh1* promoter (−8946 to −8834) were quantitated by Real-time PCR from chromatin fractions immunoprecipitated with anti-SOHLH1 antibody, anti-SP1 antibody, or control rabbit IgG. Fold enrichment represents the quantity of the region immunoprecipitated with anti-SOHLH1 (A) or anti-SP1 (B) relative to that immunoprecipitated with control rabbit IgG. Values are expressed as means ±S.E.M. of three technical replicates. *P* values were calculated by Student's *t*-test. *P<0.03.

To examine whether SOHLH1 and SP1 are recruited to the *Sohlh1* promoter region *in vivo*, we performed ChIP assay using P8 testes (**Figure 7**). By qPCR, the *Sohlh1* promoter region (−371 to −284) was shown to be significantly enriched in SOHLH1- and SP1-immunoprecipitated chromatin fractions, while a control region far upstream of the *Sohlh1* promoter (−8946 to −8834) was not. These results indicated that the sequences located immediately upstream of the *Sohlh1* transcription start site are bound by SOHLH1 and SP-1 *in vivo*.

## Discussion

In our previous study, we reported that abnormalities in the testes and ovaries of *Sohlh2*-null mice are similar to those seen in *Sohlh1*-null mice, and that *Sohlh1* transcription is downregulated in the gonads of *Sohlh2*-null mice [6]. We also demonstrated that SOHLH2 can form a heterodimer with SOHLH1 [6]. In the current study, we showed that SOHLH2 and SOHLH1 could also form homodimers (**Figure 1C, D**). We further demonstrated that SOHLH2 and SOHLH1, expressed together, upregulated the *Sohlh1* promoter through its E-boxes, while SOHLH2 or SOHLH1 alone upregulated this promoter activity only weakly (**Figure 2A**).

Similar observations have been made for MAX and MYC. MAX, a MYC-family bHLH protein, not only forms homodimers, but also forms heterodimers, preferentially with MYC [21], [22]. The MAX/MYC heterodimer binds to the E-box sequence CACGTG with higher affinity than does MAX or MYC alone [21]. MAX homodimers have no transcriptional activity, but the MAX/MYC heterodimer is a principal transcriptional activator [10], [11]. In this respect, SOHLH2 and SOHLH1 appear to function similarly to MAX and MYC.

**Figure 8:**
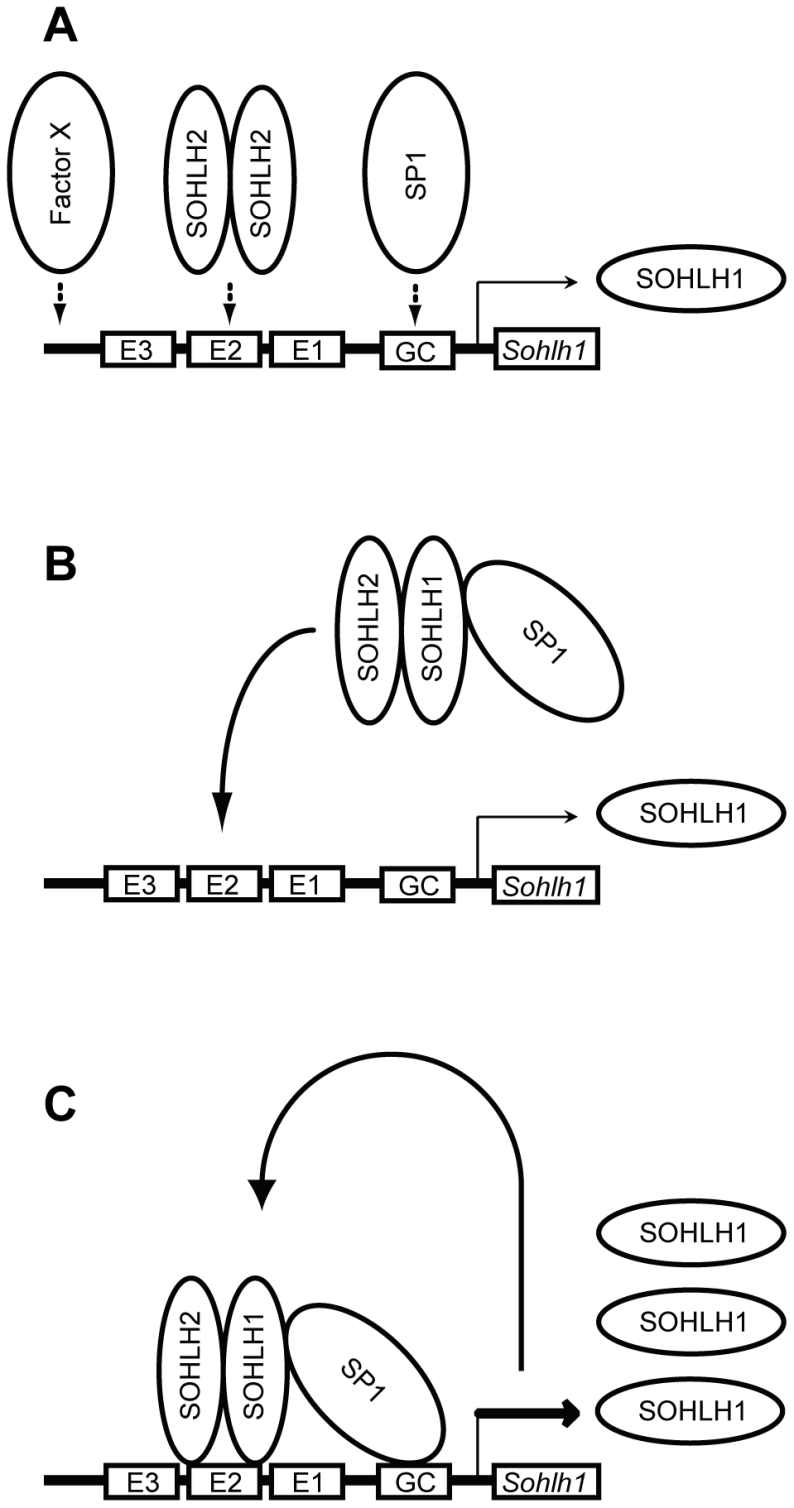
A model of *Sohlh1* gene regulation by the SOHLH2/SOHLH1/SP1 complex. (A) The SOHLH2 homodimer, SP1, or an unknown Factor X turns on weak *Sohlh1* transcription. (B) The resulting small amounts of SOHLH1 form the SOHLH2/SOHLH1/SP1 complex due to affinity. This complex is recruited to the E- and GC-box regions in the *Sohlh1* promoter. (C) The *Sohlh1* gene is highly upregulated through its auto-regulatory mechanism, which involves the SOHLH2/SOHLH1/SP1 complex.

We next demonstrated that the SOHLH proteins and SP1, which are all present in germ cells, are functionally linked. A species-conserved GC-box, which contains the SP1 consensus binding sequence, is adjacent to the *Sohlh1* promoter's E-boxes (**Figure 3**). Some bHLH proteins, including NEUROD1, interact with SP1 to synergistically activate target genes containing juxtaposed E- and GC-boxes [14], [15], [23]. It has been reported that the synergy between E12/NEUROD1 and SP1 occurs when the bHLH proteins recruit SP1 and stabilize its DNA-binding [15]. The association we observed between SOHLH1 and SP1 suggests that the SOHLH2/SOHLH1 heterodimer might also recruit SP1 and stabilize SP1's binding to the GC-box, thereby synergistically activating the *Sohlh1* promoter activity to its greatest extent (**Figure 8**). Consistent with this notion, ChIP assay using P8 testes showed that SOHLH1 and SP1 bind to the *Sohlh1* promoter region *in vivo* (**Figure 7**).

Of the three E-boxes (E1, E2, and E3), the reporter activity was reduced most greatly by a mutation in E3 (**Figure 4**). The ternary complex of NEUROD1, E12, and SP1 requires proper spacing between the E- and GC-boxes for the strongest promoter activation [15]. The SOHLH2/SOHLH1/SP1 complex is also likely to require a particular spacing between the E- and GC-boxes for maximum promoter activation, with the E3 E-box being at the most appropriate distance from the GC-box for promoter activation by the SOHLH2/SOHLH1/SP1 ternary complex.

In early spermatogenesis and oogenesis, the *Sohlh2* gene is upregulated prior to the *Sohlh1* gene [6]. However, SOHLH2 was barely able to transactivate the *Sohlh1* promoter in the absence of SOHLH1 (**Figure 2A**), so *Sohlh2* upregulation alone may not lead to *Sohlh1*'s transcription *in vivo*. The mechanism that initially activates *Sohlh1*'s transcription remains unknown. It is possible that SOHLH2 homodimers have weak transcriptional activity. Similarly, although MAX homodimers are generally thought to repress transcription, they have been found to activate transcription at low levels in a yeast system [24]. Another possibility is that other factors are responsible for turning on *Sohlh1*'s transcription (**Figure 8A**). Since *Sohlh1* transcription is detected in the gonads of *Sohlh2*-null mice [6], the *Sp1* transcript was consistently observed throughout spermatogenesis and oogenesis (data not shown), and SP1 alone was able to activate weak but detectable *Sohlh1* transcription (**Figure 5A**), SP1 might be an initiation factor for *Sohlh1* transcription (**Figure 8A**).

In *Sohlh2*-null mice, *Kit* expression is downregulated in both the testis and ovary, and KIT-positive germ-cell differentiation is disturbed [6]. In *Sohlh1*-null mice as well, KIT-positive germ-cell differentiation appears to be disturbed in the testis and ovary [5], [8]. KIT is expressed in A_diff_ spermatogonia and in primordial-to-growing oocytes, corresponding to the SOHLH2 and SOHLH1 expression. SP1 is also expressed in spermatogonia [16] and oocytes [17]. Recently, Barrios et al. [25] reported that SOHLH2 and SOHLH1 control the *Kit* expression during postnatal male germ-cell development. The *Kit* proximal promoter is reported to contain E- and GC-boxes [26], [27]. These reports suggest that the SOHLH2/SOHLH1/SP1 complex might directly regulate expression of the *Kit* gene *in vivo* through its E- and GC-boxes.

Bioinformatics analyses have predicted that a number of spermatogonia-related genes contain E- and/or GC-boxes in their 5′-cis regulatory elements [28]. The SOHLH2/SOHLH1/SP1 ternary complex might be a key factor in this transcriptional cascade. The self-regulated activity of the *Sohlh1* promoter and the synergistic action of SOHLH2, SOHLH1, and SP1 could promote rapid germ-cell differentiation (**Figure 8**). It was recently shown that retinoic acid and BMP4 are commonly involved in both early spermatogenesis and oogenesis [29]–[32], and that retinoic acid signaling directly cooperates with SP1 [33]. Further investigation of the interactions among these transcription factors and their signaling pathways should deepen our understanding of the common differentiation mechanisms of early spermatogenesis and oogenesis.
